# CDKL1 potentiates the antitumor efficacy of radioimmunotherapy by binding to transcription factor YBX1 and blocking PD-L1 expression in lung cancer

**DOI:** 10.1186/s13046-024-03007-w

**Published:** 2024-03-22

**Authors:** Zixuan Li, Huichan Xue, Jinsong Li, Zhikun Zheng, Zhiwei Liu, Xiaorong Dong, Hongbo Wang, Jing Chen, Shuangbing Xu

**Affiliations:** 1 Cancer Center, Union Hospital, Tongji Medical College, Huazhong University of Science and Technology, Wuhan, 430022 China; 2 Hubei Key Laboratory of Precision Radiation Oncology, Wuhan, 430022 China; 3 Department of Thoracic Surgery, Union Hospital, Tongji Medical College, Huazhong University of Science and Technology, Wuhan, 430022 China; 4 Clinical Research Center of Cancer Immunotherapy, Wuhan, 430022 China; 5 Institute of Radiation Oncology, Union Hospital, Tongji Medical College, Huazhong University of Science and Technology, Wuhan, 430022 China

**Keywords:** Radioimmunotherapy, Lung cancer, CDKL1, YBX1, PD-L1

## Abstract

**Background:**

The evasion of the immune response by tumor cells through programmed death-ligand 1 (PD-L1) has been identified as a factor contributing to resistance to radioimmunotherapy in lung cancer patients. However, the precise molecular mechanisms underlying the regulation of PD-L1 remain incompletely understood. This study aimed to investigate the role of cyclin-dependent kinase-like 1 (CDKL1) in the modulation of PD-L1 expression and the response to radioimmunotherapy in lung cancer.

**Methods:**

The tumorigenic roles of CDKL1 were assessed via cell growth, colony formation, and EdU assays and an in vivo nude mouse xenograft model. The in vitro radiosensitization effect of CDKL1 was evaluated using a neutral comet assay, γH2AX foci formation analysis, and a clonogenic cell survival assay. The protein‒protein interactions were confirmed via coimmunoprecipitation and GST pulldown assays. The regulation of PD-L1 by CDKL1 was evaluated via chromatin immunoprecipitation (ChIP), real-time quantitative PCR, and flow cytometry analysis. An in vitro conditioned culture model and an in vivo C57BL/6J mouse xenograft model were developed to detect the activation markers of CD8^+^ T cells and evaluate the efficacy of CDKL1 overexpression combined with radiotherapy (RT) and an anti-PD-L1 antibody in treating lung cancer.

**Results:**

CDKL1 was downregulated and suppressed the growth and proliferation of lung cancer cells and increased radiosensitivity in vitro and in vivo. Mechanistically, CDKL1 interacted with the transcription factor YBX1 and decreased the binding affinity of YBX1 for the *PD-L1* gene promoter, which consequently inhibits the expression of PD-L1, ultimately leading to the activation of CD8^+^ T cells and the inhibition of immune evasion in lung cancer. Moreover, the combination of CDKL1 overexpression, RT, and anti-PD-L1 antibody therapy exhibited the most potent antitumor efficacy against lung cancer.

**Conclusions:**

Our findings demonstrate that CDKL1 plays a crucial role in regulating PD-L1 expression, thereby enhancing the antitumor effects of radioimmunotherapy. These results suggest that CDKL1 may be a promising therapeutic target for the treatment of lung cancer.

**Supplementary information:**

The online version contains supplementary material available at 10.1186/s13046-024-03007-w.

## Introduction

Lung cancer is globally recognized as the leading cause of cancer-related death and has the highest mortality rate among cancers [1, 2]. The main treatment options for lung cancer include surgical intervention, radiotherapy (RT), chemotherapy, targeted therapy, and immunotherapy [3]. Among these modalities, checkpoint blockade immunotherapies, specifically those targeting programmed cell death protein 1 (PD-1) and programmed cell death protein 1 ligand 1 (PD-L1), have been proven to provide long-term clinical benefits to patients with non-small cell lung cancer (NSCLC) [4, 5]. Furthermore, localized RT has the potential to induce a shift from an immunologically cold tumor phenotype to a hot tumor phenotype, thereby increasing the impact of immunotherapy [6]. However, the clinical outcomes of combining immunotherapy with RT remain unsatisfactory. The abscopal response rate in patients with metastatic non-small cell lung cancer treated with pembrolizumab plus RT is 41.7% [7, 8]. Extensive evidence indicates that the expression level of tumor PD-L1 serves as a crucial biomarker for evaluating the clinical response of lung cancer patients to anti-PD-1/PD-L1 therapy [9]. Consequently, exploring the mechanisms of PD-L1 regulation is highly important for improving the response to lung cancer immunotherapy.

Cyclin-dependent kinases (CDKs) are a group of serine-threonine protein kinases that play a crucial role in the cell cycle process by interacting with cyclins to regulate various checkpoints, such as G1/S and G2/M [10, 11]. In human cells, more than 20 CDKs have been identified and categorized into two main classes. CDK1, CDK2, and CDK4-6 are primarily involved in cell cycle control, while CDK7-9, CDK11, and CDK12 are associated with gene transcription [12, 13]. CDKs are essential for regulating cell metabolism, metastasis, cancer immunity, inflammation, and DNA damage repair [14, 15]. For instance, inhibition of the CDK4-cyclin D complex has been shown to improve the effectiveness of immunotherapy by regulating the abundance of the PD-L1 protein through proteasome-mediated ubiquitination [16]. Cyclin-dependent kinases like 1 (CDKL1), a member of the CDKL family, has high sequence homology with CDKs and possesses multiple binding sites for MAPK, suggesting that it can selectively mediate protein interactions [17]. CDKL1 is expressed in the lungs, brain and ovaries and is implicated in processes such as cilia formation, cilium length regulation, brain development, and cancer progression [18–20]. However, the specific role of CDKL1 in regulating the response to RT and immunotherapy in lung cancer remains largely unexplored.

Y box binding protein 1 (YBX1, also known as YB1), a member of the cold-shock domain protein family, is primarily localized in the cytoplasm but can translocate to the nucleus and bind to target gene promoters in response to various stimuli [21, 22]. Furthermore, YBX1 also functions as an RNA binding protein, influencing mRNA transcription, translation, and stability [23]. YBX1 plays an important role in multiple biological processes within tumor cells, including the regulation of tumor immunity [24, 25]. In the context of breast cancer, YBX1 facilitates cell proliferation and metastasis by activating the PD-1/PD-L1 pathway [26]. Furthermore, research has demonstrated that chemoresistant tumor cells can promote the binding of YBX1 to the PD-L1 promoter region, thereby promoting the transcription of PD-L1 and facilitating immune evasion in liver cancer [27]. These findings collectively suggest that the YBX1/PD-L1 axis actively contributes to the immune modulation of tumor cells.

In this study, we provide evidence that the overexpression of CDKL1 suppresses tumorigenesis and enhances radiosensitivity in lung cancer. Moreover, we demonstrated that CDKL1 interacts with YBX1 and negatively regulates the expression of PD-L1, thereby promoting the activation of CD8^+^ T cells. Importantly, the combination of CDKL1 overexpression with RT and anti-PD-L1 antibody treatment elicits a potent immune response against tumor cells in lung cancer.

## Materials and methods

### Cell lines and transfection

The cell lines utilized in this study were obtained from the American Type Culture Collection (ATCC) and included human lung cancer cells (A549, H1299, H460, and H522), a human bronchial epithelioid cell line (BEAS-2B), HEK293T cells, and a mouse Lewis cell line. These cell lines were cultured in DMEM or RPMI-1640 medium at 37 °C under 5% CO_2_. SiRNA transfection was conducted using the transfection reagent Lipofectamine RNAiMAX (Invitrogen) for 48 h. The specific siRNA sequences used in this experiment were as follows:

si-CDKL1-1: 5’-GCAAGUGUUUAGCACGAAU-3’;

si-CDKL1-2: 5’-GUGAUACCAAGAAACUUAA-3’; and.

si-YBX1: 5’-UACAUCUUCCUUGGUGUCA-3’.

### Establishment of stable CDKL1-overexpressing cells

As described in our previous study [28], HEK293T cells were cultured at a density of 30–50% and subsequently transfected with the viral packaging plasmids pSPAX2 and pMD2 and Flag-tagged CDKL1. After 48 h, the cell culture supernatant was collected and filtered. Lentivirus supernatants were then used to infect A549 and Lewis cells in the presence of polybrene (10 µg/mL). The stable cells were selected using puromycin (2 µg/mL), and confirmed by western blotting.

### Construction of an in vitro conditioned culture model

This method was performed as previously described [29]. In brief, T cells were obtained from the spleens of C57BL/6J mice, and CD8^+^ T cells were isolated using the Mouse CD8 + T-Cell Isolation Kit (BioLegend, 480,008) according to the manufacturer’s instructions. The isolated CD8^+^ T cells were then cultured with Lewis cells and stimulated with IL-2 and anti-CD3/CD28 antibodies. Functional experiments were performed three days later.

### Real-time quantitative PCR (qPCR)

RNA extraction was carried out at 4 °C using the Total RNA Kit I (Omega, R6834-01), and then reverse transcription was performed using ABScript III RT Master Mix for qPCR with gDNA Remover (ABclonal, RK20429). The cells were lysed using lysis buffer, and the RNA was subsequently centrifuged at 10,000 rpm for 2 min to separate it from the cells. Each experimental group was independently assessed three times. The sequences of primers used in this study are listed in Supplementary Table 1.

### Western blotting

Cells were lysed with NETN buffer on ice for 20 min and then centrifuged at 12,000 rpm for 20 min to extract proteins. Then, the proteins were separated by SDS-PAGE. The following antibodies were used: anti-CDKL1 antibody (Abcam, ab136129), anti-YBX1 antibody (Proteintech, 20339-1-AP), anti-GAPDH antibody (Proteintech, 60,004-I-Ig), anti-Flag-tag antibody (ABclonal, AE005), anti-Myc-tag antibody (ABclonal, AE010), anti-H3 antibody (Proteintech, 17168-1-AP), anti-α-tubulin antibody (Proteintech, 11224-1-AP), and anti-PD-L1 antibody (Proteintech, 17952-1-AP).

### Coimmunoprecipitation (co-IP) assay

To assess exogenous binding, the Flag-tag and Myc-tag plasmids were introduced into HEK293T cells, and the cell extracts were incubated with streptavidin (S) beads (Millipore, 3,761,230) overnight at 4 °C. To assess endogenous interactions, the cell extracts were incubated with protein A/G agarose (Santa Cruz, C0620) along with either IgG or YBX1 antibody (Proteintech, 20339-1-AP). Subsequently, the protein samples were subjected to five rounds of washing using NETN buffer prior to western blotting.

### GST pulldown assay

The GST-only and GST-CDKL1 fusion proteins were purified from *Escherichia coli* and subsequently incubated with the SFB-YBX1 protein and GST beads overnight. Next, the samples were washed five times and subjected to SDS‒PAGE.

### ChIP-PCR

A ChIP Assay Kit (Beyotime, P2078) was used in accordance with the manufacturer’s instructions. Briefly, a 1% formaldehyde solution was used to cross-link the cells in each group. The cells were then collected and resuspended in SDS lysis buffer before being sonicated. Then, the suspension was centrifuged, and the resulting supernatant was combined with Chip dilution buffer. Agarose and either an anti-YBX1 antibody (Proteintech, 20339-1-AP) or an anti-IgG antibody were added, followed by overnight incubation at 4 °C. The proteins were subsequently eluted from the beads after washing. The samples were then subjected to heating at 65 °C for 4 h. Finally, the changes in YBX1 accumulation at the PD-L1 promoter were assessed using qPCR, and the primer sequences are provided in Supplementary Table 2.

### EdU assay

An EdU kit (Beyotime, C0078S) was used to assess cell proliferation. Following the overexpression or knockdown of CDKL1, cells were seeded at a density of 4 × 10^4^ cells per well for 24 h. Subsequently, the cells were incubated with EdU (10 µM) at 37 °C for 2 h. After fixation, washing, and permeabilization, the cells were incubated in a click reaction solution for 30 min, followed by staining of the nucleus. The results were analyzed using a fluorescence microscope.

### Neutral comet assay

A comet assay kit (4250-050-k) was utilized for this experimental procedure. The cells were digested 4 h after irradiation. The cell suspension was then mixed with low-melting-point agarose (LMA) and placed onto an electrophoresis plate. After 20 min, the plate was immersed in precooled lysis buffer at 4 °C for 1 h. Electrophoresis was conducted at a current of 21 mA for 1 h. The gel was subsequently immersed in a DNA precipitation solution for 30 min. The cells were stained with SYBR green dye for 8 min and left to dry completely overnight.

### Immunofluorescence staining

The cells were transfected with the specified siRNAs and subsequently exposed to irradiation. Four hours post irradiation (6 Gy), the cells were fixed, permeabilized, and incubated with an anti-γH2AX antibody (Abcam, ab26350) at 4 °C overnight. Subsequently, the cells were incubated with anti-DyLight 549 (Abbkine, A23310) at 37 °C for 50 min, and nuclear staining was carried out using DAPI.

### Clonogenic cell survival assay

Cells at different densities were exposed to varying doses of irradiation (0, 2, 4, 6, and 8 Gy). After two weeks, the cell colonies were fixed and subjected to crystal violet staining. Colonies consisting of more than 50 cells were evaluated.

### Flow cytometry analysis

In the in vitro experiments, cells were cultured in 6-well plates and subsequently digested and washed before staining. In the in vivo experiments, single-cell suspensions were obtained from the tumor tissue after crushing and filtering the tissue. The cells were then subjected to staining with the corresponding antibodies on ice for 30 min in the dark. The reagents employed for staining included Zombie Aqua (423,102) for labeling dead cells and anti-CD8 (100,706), anti-CD45 (103,154), anti-IFN-γ (505,826), and anti-GZMB antibodies (372,204) for labeling T-cell markers. All antibodies utilized for flow cytometry were procured from BioLegend.

### Mouse xenograft models and in vivo treatments

Xenograft models have been described in detail previously [29]. Briefly, the mice were randomly divided into the indicated groups. Tumor volume was assessed at regular intervals of three days using the formula V = L × W^2^/2. In the case of RT, the nude mice were subjected to a single dose of 10 Gy, while the C57BL/6J mice received three fractions of 8 Gy each. In the CDKL1-overexpressing group, nude mice were injected with 5 × 10^6^ A549 stable cells, whereas C57/BL6J mice were injected with 8 × 10^5^ Lewis stable cells. To block PD-L1 expression and CD8^+^ T cell activation, an anti-PD-L1 antibody (BioXcell, BE0101) or anti-CD8^+^ T-cell antibody (BioXcell, BP0061) was administered via injection every four days.

### Statistical analysis

The tail moment in the comet assay was analyzed using CometScore software. Statistical analysis of all the experimental results was performed using GraphPad Prism 8 with Student’s t test. A significance level of *P* < 0.05 indicated a significant difference (* *P* < 0.05, ** *P* < 0.01, *** *P* < 0.001). All experiments were independently repeated at least three times.

## Results

### CDKL1 is lowly expressed and inhibits the growth and proliferation of lung cancer cells

**Fig. 1:**
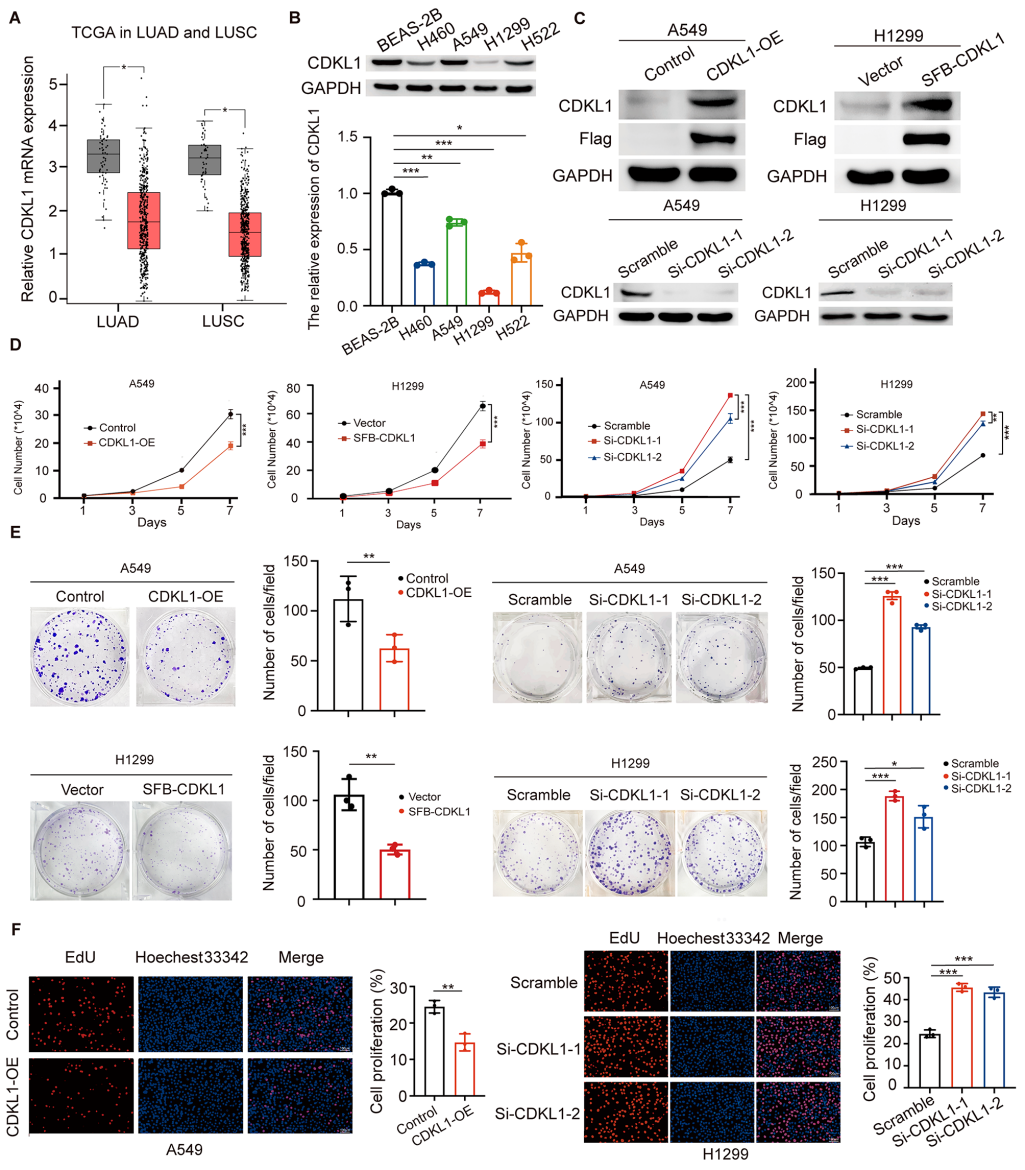
CDKL1 is lowly expressed and inhibits the growth and proliferation of lung cancer cells. (**A**) The TCGA database was utilized to predict the mRNA levels of *CDKL1* in both LUAD (*N* = 59, T = 483) and LUSC patients (*N* = 50, T = 486). * *P* < 0.05. (**B**) The expression of CDKL1 in various lung cancer cell lines was assessed by western blot analysis. The grey values of the bands were quantitatively analyzed, and the expression level of CDKL1 was normalized to that of GAPDH (* *P* < 0.05, ** *P* < 0.01, *** *P* < 0.001). (**C**) Western blot analysis was used to assess CDKL1 protein expression in A549 and H1299 cells with CDKL1 overexpression or knockdown. In lung cancer cell lines with CDKL1 overexpression, CDKL1 protein levels were assessed using CDKL1 or Flag antibodies. (**D**) Cell growth ability was examined by cell growth curves every other day in A549 and H1299 cells with either CDKL1 overexpression or knockdown (** *P* < 0.01, *** *P* < 0.001). (**E**) A549 and H1299 cells with CDKL1 overexpression or knockdown were seeded into 6-well plates at a density of 500 cells per well. After two weeks, the number of clones (> 50 cells) was determined (** *P* < 0.01, *** *P* < 0.001). (**F**) The proliferation ability of the A549 and H1299 cells was evaluated using the EdU assay. The percentage of EdU-positive cells was calculated (** *P* < 0.01, *** *P* < 0.001). Scale bar, 100 μm

To investigate the role of CDKL1 in lung cancer, an analysis of mRNA expression was conducted using lung adenocarcinoma (LUAD) and lung squamous cell carcinoma (LUSC) datasets from the TCGA database. The results indicated that *CDKL1* mRNA levels were lower in LUAD and LUSC tissues than in normal lung tissues (Fig. 1A). Subsequently, the protein levels of CDKL1 were assessed in five cell lines (BEAS-2B, H460, A549, H1299, and H522). As shown in Fig. 1B, the expression of CDKL1 in all lung cancer cell lines was lower than that in human bronchial epithelioid cells (BEAS-2B). Next, we stably overexpressed CDKL1 or knocked down CDKL1 in A549 and H1299 cells, respectively, and subsequently confirmed the efficacy of both CDKL1 overexpression and knockdown via western blot analysis (Fig. 1C). The observed overexpression of CDKL1 resulted in notable inhibition of lung cancer cell growth and a decrease in colony formation ability in vitro (Fig. 1D, E). Conversely, CDKL1 silencing accelerated the growth and proliferation of lung cancer cells (Fig. 1D, E). Consistent results were observed in A549 and H1299 cells when CDKL1 was either overexpressed or knocked down. The abovementioned findings were further supported by the results of the EdU assay, which showed a decrease in the percentage of EdU-positive CDKL1-overexpressing cells and the opposite effect in cells with CDKL1 depletion (Fig. 1F). Collectively, these results suggest that CDKL1 may act as a tumor suppressor protein in lung cancer.

### CDKL1 overexpression enhances the radiosensitivity of lung cancer in vitro and in vivo

**Fig. 2:**
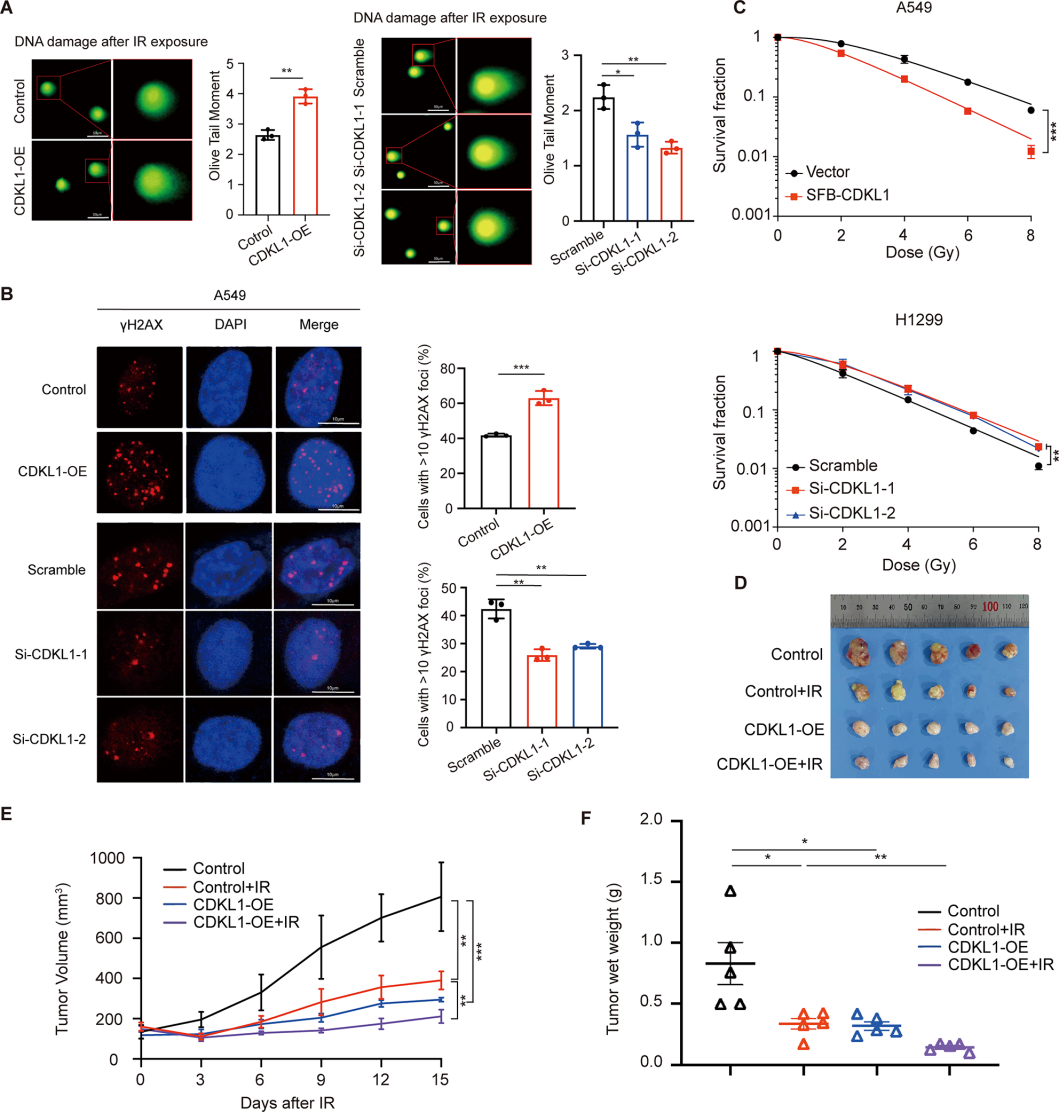
CDKL1 overexpression increases the radiosensitivity of lung cancer in vitro and in vivo. (**A**) CDKL1 was overexpressed in A549 cells and downregulated using siRNA in H1299 cells. The comet assay was conducted 4 h after irradiating the cells with 6 Gy. The tail moment of the comet assay was quantified (* *P* < 0.05, ** *P* < 0.01). Scale bar, 50 μm. (**B**) Representative immunostaining images of γH2AX in A549 cells with CDKL1 overexpression and si-CDKL1 treatment are shown. The number of γH2AX-positive cells (containing 10 or more foci) was determined and statistically analyzed 4 h after 6 Gy irradiation (** *P* < 0.01, *** *P* < 0.001). Scale bar, 10 μm. (**C**) The number of colonies formed was quantified after 14 days of irradiation (** *P* < 0.01, *** *P* < 0.001). (**D**) Images of the xenograft tumors in nude mice (*n* = 5). (**E**) Tumor volume was recorded every three days (*n* = 5). (**F**) The wet weight of the tumors was also determined (*n* = 5)

To further investigate the role of CDKL1 in the response to DNA damage in lung cancer, we initially conducted a neutral comet assay. The results revealed that overexpression of CDKL1 led to an increase in the tail moment of the comet, while depletion of CDKL1 had the opposite effect (Fig. 2A). It has been previously reported that DNA damage can induce phosphorylation at the serine 139 site of histone H2AX, resulting in the formation of γH2AX at the DNA damage site [30]. Hence, we examined the formation of γH2AX foci and observed a notable increase in the number of γH2AX foci in cells overexpressing CDKL1, while a decrease in the number of γH2AX foci was observed in CDKL1-knockdown cells following exposure to ionizing radiation (IR) (Fig. 2B). These findings suggest that CDKL1 enhances the DNA damage response (DDR).

An aberrant DNA damage response is a crucial contributor to the development of radiation resistance in tumor cells. As expected, the overexpression of CDKL1 significantly increased cellular sensitivity to irradiation, whereas the depletion of CDKL1 conferred radioresistance in lung cancer cells (Fig. 2C). To investigate the potential role of CDKL1 in facilitating radiosensitization in vivo, we generated A549 cells that overexpress CDKL1 and subsequently established a subcutaneous transplanted tumor model in nude mice. As shown in Fig. 2D-F, both CDKL1 overexpression and radiation treatment alone significantly impeded tumor growth and reduced the weight of the xenograft tumors, indicating that CDKL1 does indeed suppress tumorigenesis in lung cancer in vivo. Furthermore, tumors that overexpressed CDKL1 in combination with IR exhibited slower growth than those treated with radiation therapy alone. These findings support the notion that CDKL1 increases the radiosensitivity of lung cancer in vitro and in vivo.

### CDKL1 interacts with the transcription factor YBX1 in vitro and in vivo

**Fig. 3:**
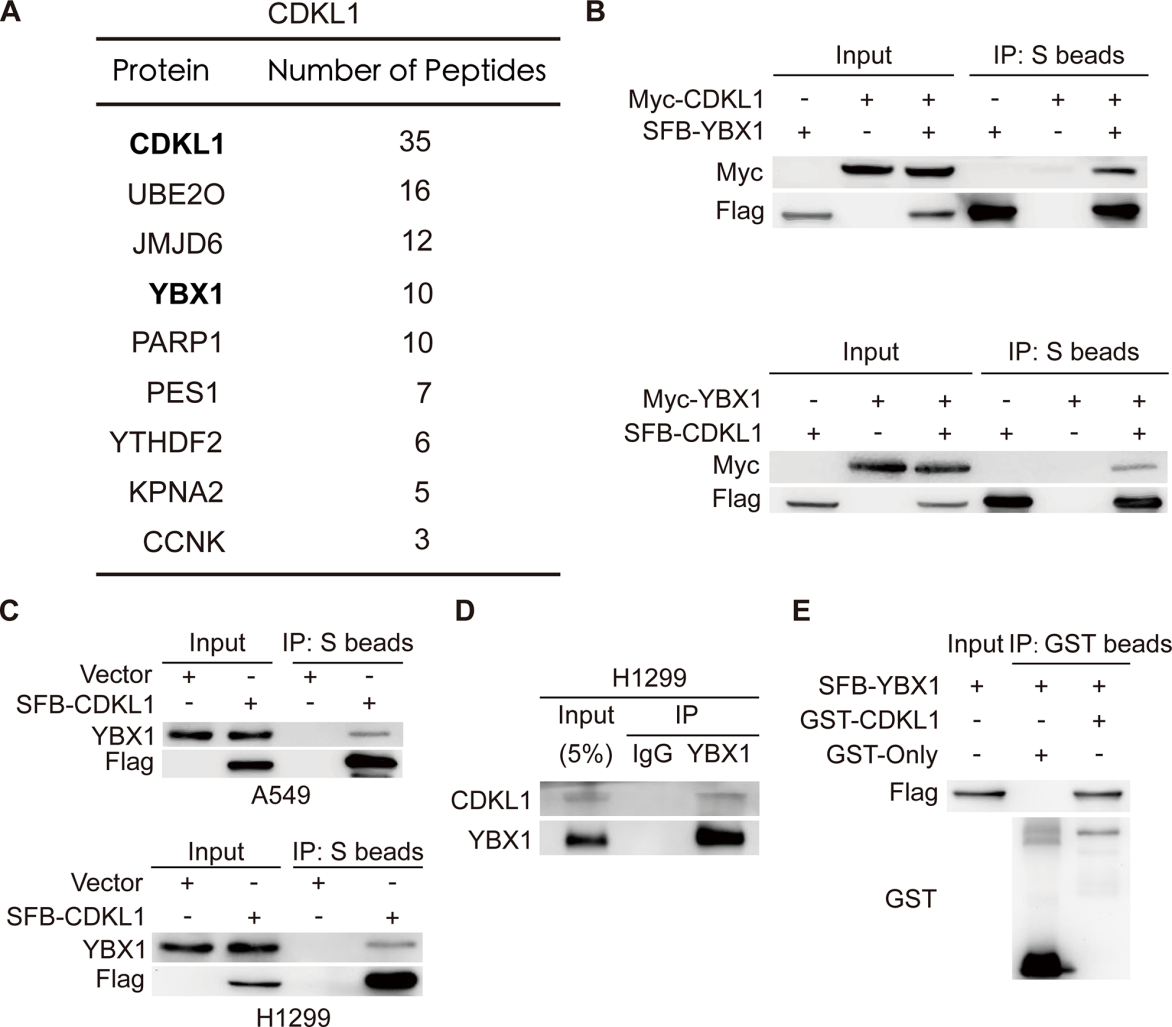
CDKL1 interacts with YBX1 in vitro and in vivo. (**A**) The proteins that interact with CDKL1 were identified by screening using tandem affinity purification (TAP) and mass spectrometry (MS). (**B**) The interaction between exogenous CDKL1 and exogenous YBX1 was assessed in HEK293T cells. (**C**) The interaction between exogenous CDKL1 and endogenous YBX1 was assessed in A549 and H1299 cells. (**D**) In H1299 cells, the endogenous interaction between CDKL1 and YBX1 was examined using anti-IgG and anti-YBX1 antibodies. (**E**) GST-only and GST-CDKL1 proteins were isolated from bacteria. Proteins extracted from HEK293T cells transfected with SFB-YBX1 were incubated with GST-only or GST-CDKL1 overnight, and their presence was detected by immunoblotting

To elucidate the molecular mechanisms underlying the functions of CDKL1 in lung cancer, proteomic screening was previously conducted using tandem affinity purification (TAP) and mass spectrometry (MS), which revealed that the transcription factor YBX1 is a potential interacting partner of CDKL1 (Fig. 3A) [31]. To confirm this association, a coimmunoprecipitation experiment was conducted. As shown in Fig. 3B, exogenously expressed CDKL1 interacted with exogenously expressed YBX1, and vice versa. Exogenous CDKL1 was also found to interact with endogenous YBX1 in both the A549 and H1299 cell lines (Fig. 3C). Notably, endogenous CDKL1 was observed to bind to endogenous YBX1 in lung cancer cells (Fig. 3D). This in vitro binding was further supported by the results of a GST pulldown assay, which confirmed a direct interaction between CDKL1 and YBX1 (Fig. 3E). These findings confirm the proteomic results and demonstrate a physical interaction between CDKL1 and YBX1 in lung cancer.

### CDKL1 overexpression downregulates PD-L1 in a YBX1-dependent manner in lung cancer

**Fig. 4:**
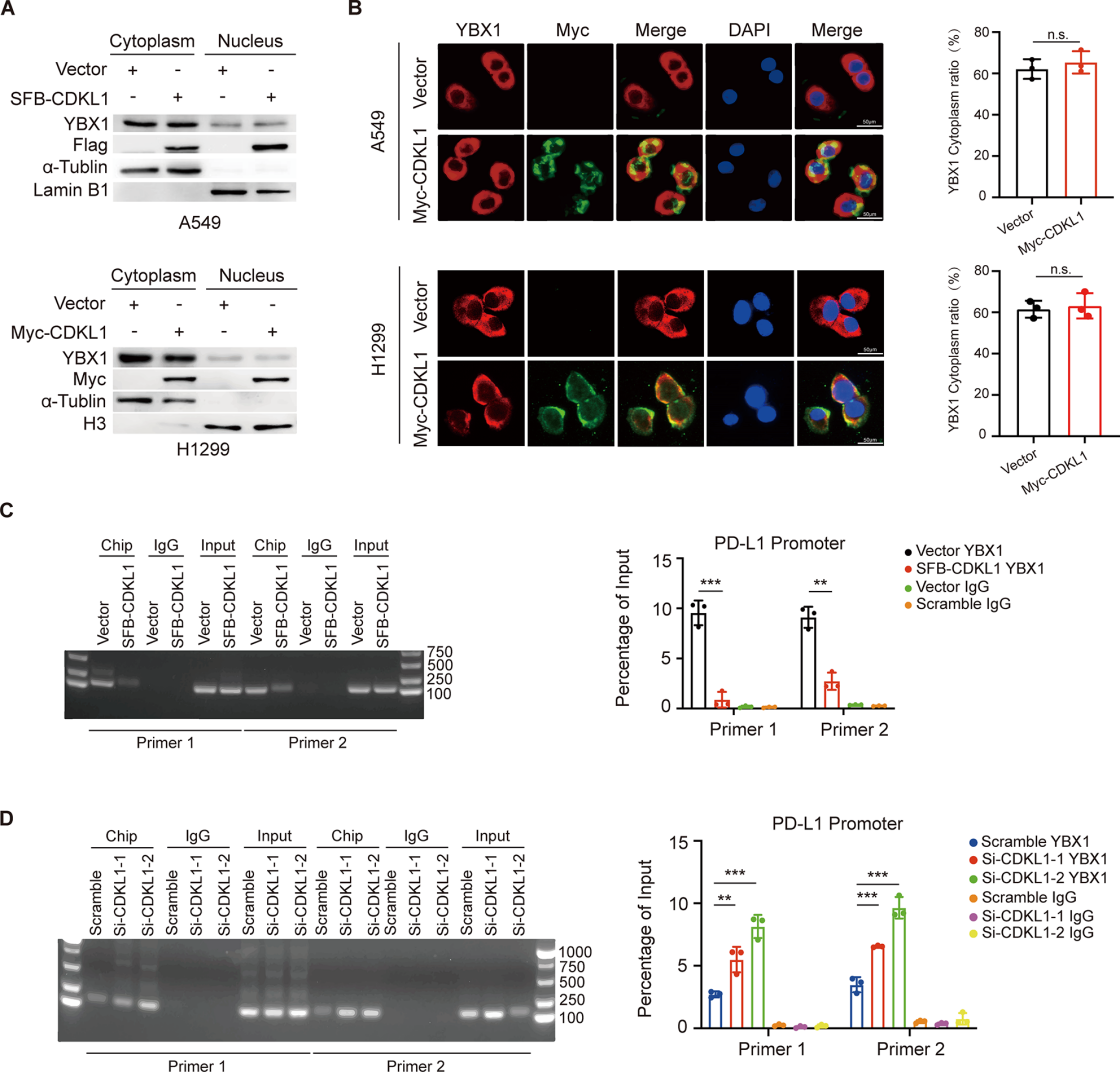
CDKL1 overexpression inhibits YBX1 accumulation at the PD-L1 promoter but does not affect YBX1 localization. (**A**) The expression of YBX1 in the cytoplasm and nucleus was assessed in A549 and H1299 cells overexpressing CDKL1. (**B**) The nuclear and cytoplasmic localization of YBX1 was detected by immunofluorescence staining, and statistical analysis was performed (n.s. indicates *P* > 0.05). Scale bar, 50 μm. (**C**) The gel electrophoresis results of H1299 cells transfected with SFB-CDKL1 are presented as representative images. The levels of YBX1 at the PD-L1 promoter were normalized to the concentration of input DNA (** *P* < 0.01, *** *P* < 0.001). (**D**) The gel electrophoresis results of A549 cells transfected with si-CDKL1 are shown as representative images. The levels of YBX1 at the PD-L1 promoter were normalized to the concentration of input DNA (** *P* < 0.01, *** *P* < 0.001)

**Fig. 5:**
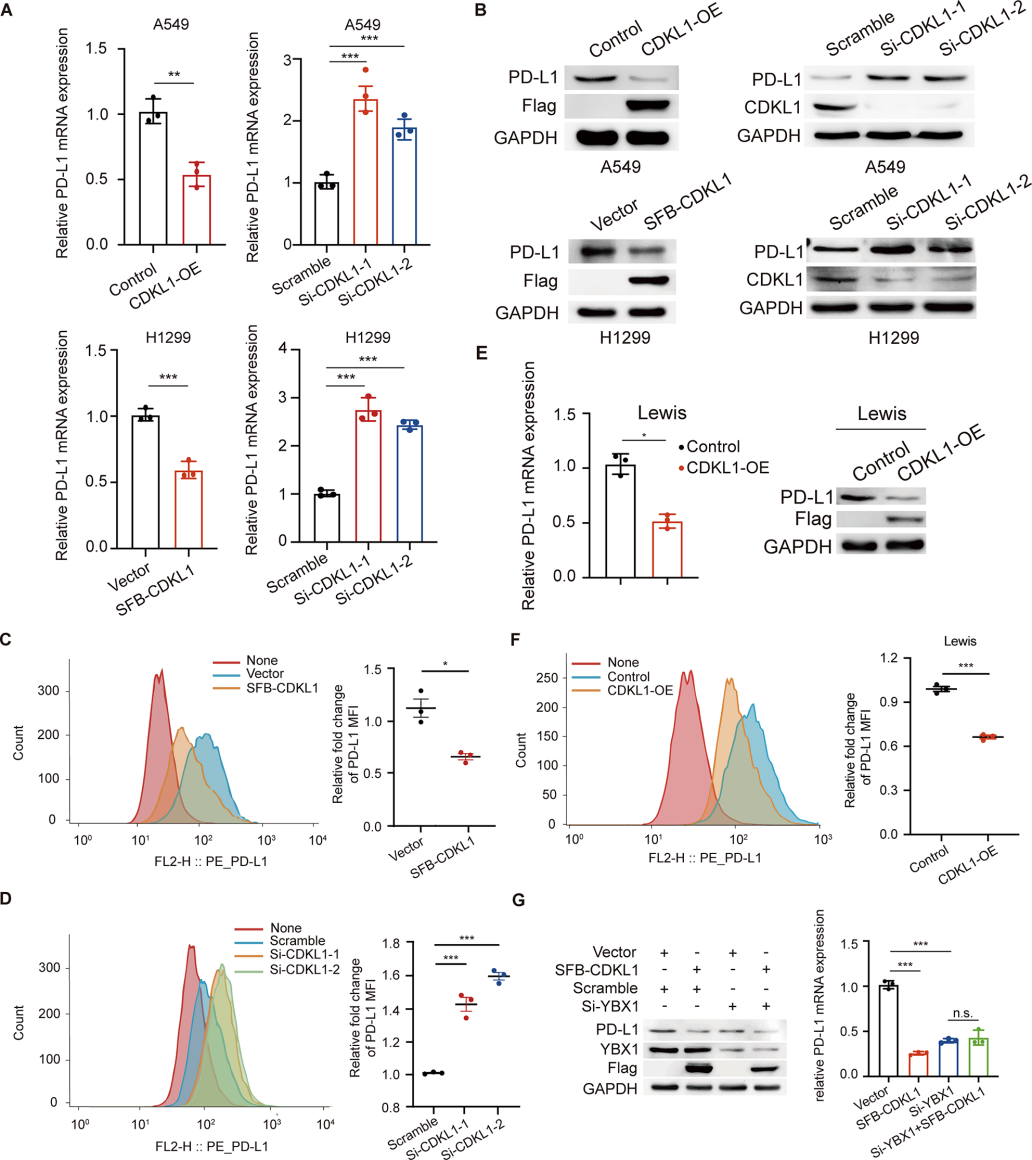
CDKL1 overexpression downregulates the expression of PD-L1 through YBX1. (**A**) The mRNA level of *PD-L1* was assessed in A549 and H1299 cells using qPCR (*** *P* < 0.001). (**B**) The protein level of PD-L1 in A549 and H1299 cells overexpressing CDKL1 or transfected with si-CDKL1 was determined using western blotting. (**C**) H1299 cells were transfected with SFB-CDKL1, and the expression of PD-L1 on the surface of living cells was determined using flow cytometry (* *P* < 0.05). (**D**) SiRNA was transfected into H1299 cells to silence CDKL1, and the expression of PD-L1 on the surface of living cells was determined using flow cytometry (*** *P* < 0.001). (**E**) The mRNA and protein levels of PD-L1 were assessed using qPCR after CDKL1 was overexpressed in Lewis cells. (**F**) The expression of PD-L1 on the cell surface of Lewis cells overexpressing CDKL1 was examined using flow cytometry (*** *P* < 0.001). (**G**) The protein and mRNA expression levels of PD-L1 were determined in A549 cells (n.s. *P* > 0.05, *** *P* < 0.001)

YBX1 is predominantly localized in the cytoplasm and translocates to the nucleus to interact with the promoter regions of specific genes, thereby initiating transcription. To determine the regulatory effect of CDKL1 on YBX1, we assessed the subcellular localization of YBX1 and revealed that CDKL1 and YBX1 colocalized in the cytoplasm and that the overexpression of CDKL1 did not impact the localization of YBX1 in either A549 or H1299 cells (Fig. 4A, B). Previous studies have reported that the recruitment of YBX1 to the binding site on the PD-L1 promoter is crucial for the transcriptional activation of the PD-L1 gene [27, 32]. Therefore, we explored whether CDKL1 affects YBX1 occupancy at the PD-L1 promoter. ChIP-PCR experiments revealed that the overexpression of CDKL1 resulted in a reduction in YBX1 binding at the PD-L1 promoter region, whereas the knockdown of CDKL1 increased YBX1 binding to the PD-L1 promoter region (Fig. 4C, D). Consistent with this idea, CDKL1 negatively regulated PD-L1 expression in two different human lung cancer cell lines (A549 and H1299) at both the mRNA and protein levels (Fig. 5A, B).

PD-L1 is known to be transported to the cell membrane following synthesis, where it exerts an antitumor immune effect. To further investigate this effect, we performed a flow cytometry assay to investigate the impact of CDKL1 on the expression of PD-L1 on the cell membrane. As shown in Fig. 5C, D, the expression of PD-L1 on the surface of viable cells was downregulated after the overexpression of CDKL1 and, conversely, was upregulated when CDKL1 was depleted. Similarly, the overexpression of CDKL1 resulted in significant downregulation of both PD-L1 mRNA and protein expression in mouse Lewis cells (Fig. 5E, F). These findings suggest that CDKL1 acts as a negative regulator of PD-L1 expression across various cell lines. To further investigate the role of YBX1 in the regulation of PD-L1 by CDKL1, we conducted rescue experiments and revealed that the overexpression of CDKL1 or the knockdown of YBX1 decreased the expression of PD-L1 (Fig. 5G). However, when CDKL1 was overexpressed in YBX1-depleted cells, there was no significant alteration in the mRNA or protein levels of PD-L1 (Fig. 5G). These findings suggest that CDKL1 negatively regulates the expression of PD-L1 in a YBX1-dependent manner in lung cancer.

### CDKL1 overexpression induces the activation of CD8^+^ T cells and promotes the antitumor immune response in lung cancer

**Fig. 6:**
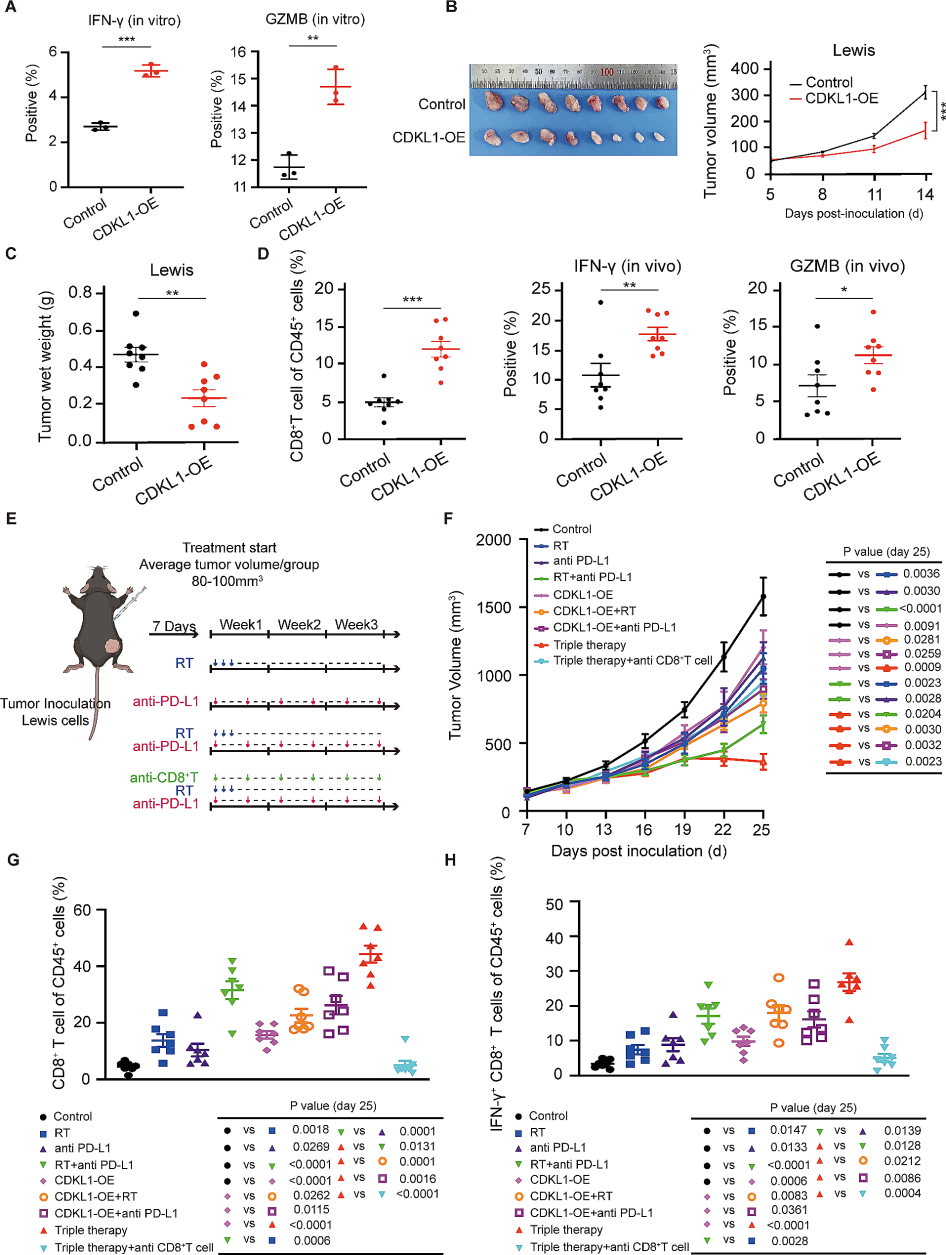
CDKL1 overexpression inhibits immune evasion in vivo and in vitro, and triple therapy yields the most effective antitumor outcomes in lung cancer. (**A**) The percentage changes in IFN-γ and GZMB levels in each group were analyzed using flow cytometry in vitro (** *P* < 0.01, *** *P* < 0.001). (**B**) Images of xenograft tumors in C57BL/6J mice. Tumors were measured for volume determination every three days (*n* = 8, *** *P* < 0.001). (**C**) Tumor wet weight was determined (*n* = 8, ** *P* < 0.01). (**D**) The percentage of CD8^+^ T cells among CD45^+^ cells, as well as the levels of IFN-γ and GZMB, were assessed in the subcutaneously transplanted tumors on day 14 after inoculation (* *P* < 0.05, ** *P* < 0.01, *** *P* < 0.001). (**E**) A schematic diagram illustrating the treatment protocol. (**F**) The mice were randomly divided into nine groups and subjected to treatment. Tumors were measured for volume determination every three days (*n* = 7). (**G**) The percentage of CD8^+^ T cells among CD45^+^ cells within the subcutaneously transplanted tumors was assessed on day 25 after treatment with various therapies (*n* = 7). (**H**) The amount of IFN-γ secreted by CD8^+^ T cells within the subcutaneously transplanted tumors was determined on day 25 post inoculation (*n* = 7)

The presence of high levels of PD-L1 on tumor cells hinders the activation of CD8^+^ T cells, thereby facilitating immune evasion by tumor cells [33, 34]. Consequently, we propose that CDKL1-mediated regulation of PD-L1 may impact the activation of CD8^+^ T cells. To validate this hypothesis, we cultured Lewis cells with CDKL1 overexpression and CD8^+^ T cells extracted from the spleens of mice. Flow cytometry clearly demonstrated a significant increase in the expression of the cytotoxicity markers IFN-γ and GZMB in CD8^+^ T cells when they were cultured with cells overexpressing CDKL1 (Fig. 6A). Subsequently, we inoculated a stable Lewis cell line overexpressing CDKL1 into C57BL/6J mice to establish a subcutaneous tumor model. The results revealed a notable deceleration in the growth rate of transplanted tumors following the overexpression of CDKL1, accompanied by a decrease in the wet weight of the tumors (Fig. 6B, C). To elucidate the role of CDKL1 in immune evasion, we ground transplanted tumors into single-cell suspensions and subsequently conducted flow cytometry staining to assess the proportion of CD8^+^ T cells and the levels of their secreted factors within the tumor. As shown in Fig. 6D, the proportion of CD8^+^ T cells among CD45 + cells was increased in tumors with CDKL1 overexpression, accompanied by an increase in the secretion of IFN-γ and GZMB. This finding suggested that CDKL1 has the potential to stimulate the activation of CD8^+^ T cells. In summary, our findings indicate that CDKL1 induces the activation of CD8^+^ T cells, thereby augmenting the antitumor immune response.

### The combination of CDKL1 overexpression, RT, and anti-PD-L1 antibody therapy induces the greatest antitumor efficacy in lung cancer

Given that the combination of overexpression of CDKL1 in nude mice with RT increased the therapeutic efficacy and CDKL1 overexpression inhibited the expression of PD-L1 while stimulating CD8^+^ T-cell activation, we hypothesized that the combination of CDKL1 overexpression, RT, and immunotherapy (anti-PD-L1 antibody) may synergistically augment the antitumor response. We performed a study in which we implemented single therapy, dual therapy, and triple therapy involving the overexpression of CDKL1, RT, and anti-PD-L1 immunotherapy (Fig. 6E). To evaluate the efficacy of these treatments, we established nine groups of C57BL/6J mice with subcutaneously transplanted tumors and assessed the antitumor effects in these groups. Our findings demonstrated that treatment with any one of the therapies alone effectively inhibited tumor growth (Fig. 6F). Compared to the control, combinations of two of these treatments had a greater ability to impede tumor growth, while triple therapy had the most substantial effect on tumor growth (Fig. 6F). Furthermore, both the monotherapy and dual combination groups had an increase in the proportion of CD8^+^ T cells and the proportion of cytotoxic IFN-γ^+^ CD8^+^ T cells, and the triple therapy group exhibited the most pronounced effect (Fig. 6G, H). Notably, the effectiveness of triple therapy was significantly reduced when CD8^+^ T cells were depleted (Fig. 6F-H), suggesting the indispensability of CD8^+^ T cells in the immune response against tumors. Collectively, these findings suggest that the combined administration of CDKL1 overexpression, RT, and anti-PD-L1 antibody therapy has the strongest antitumor effect on lung cancer.

## Discussion

This study provides evidence demonstrating that CDKL1 is expressed at low levels, inhibits tumorigenesis and enhances radiosensitivity in lung cancer. Furthermore, we identified YBX1 as a novel CDKL1-interacting protein and elucidated a new mechanism by which CDKL1 affects tumor immune evasion through the YBX1/PD-L1 axis. More importantly, we confirmed that compared to monotherapy and dual therapy, triple therapy consisting of CDKL1 overexpression, RT, and anti-PD-L1 antibody treatment has the most potent synergistic antitumor effect on lung cancer.

CDK proteins are crucial molecules involved in the regulation of the cell cycle. The overactivation or inactivation of CDKs in tumor-related signaling pathways has been implicated in the development of cancer [35]. CDKL1, which was initially discovered in the astrocytes of neuroblastoma patients, has functions comparable to those of other CDK proteins. CDKL1 is closely associated with the growth and proliferation of malignant tumors [36–38]. This study shows for the first time that CDKL1 effectively inhibits the growth and proliferation of lung cancer cells both in vitro and in vivo. Additionally, our investigation revealed the involvement of CDKL1 in the DNA damage response. The overexpression of CDKL1 resulted in an increase in the DNA damage signal and led to increased sensitivity of cells to irradiation both in vitro and in vivo. Thus, our study revealed novel biological functions of CDKL1, suggesting that CDKL1 could serve as a promising target for the treatment of lung cancer.

YBX1 plays a crucial role in regulating the growth, proliferation, metastasis, and apoptosis of tumor cells, as well as in DNA and RNA transcription [24, 25]. Previous studies have demonstrated that the tumor suppressor gene BRD7 interacts with YBX1 to facilitate its degradation and consequently inhibit epithelial–mesenchymal transition and metastasis in breast cancer [39]. Furthermore, it has been established that YBX1 plays a role in the regulation of tumor immunity [27, 32]. Specifically, YBX1-mediated upregulation of PD-L1 has been shown to facilitate immune evasion and chemoresistance in hepatocellular carcinoma (HCC) [27]. In our study, we identified CDKL1 as a novel binding partner of YBX1, and this interaction did not affect the subcellular localization of YBX1. However, CDKL1 interacts with YBX1 and suppresses the recruitment of YBX1 to the promoter region of PD-L1, thereby inhibiting PD-L1 transcription. Notably, the overexpression of CDKL1 leads to a reduction in PD-L1 expression, and this regulatory mechanism is dependent on the presence of YBX1. Hence, these findings elucidate the role of CDKL1 as an upstream negative regulator of PD-L1, revealing a novel mechanism governing the expression of PD-L1 in lung cancer.

The efficacy of immunotherapy is contingent upon CD8^+^ T-cell-mediated antitumor immunity [40]. Our study confirmed that CDKL1 overexpression induces the activation of CD8^+^ T cells both in vitro and in vivo. Furthermore, the combination of overexpression of CDKL1 and treatment with RT and an anti-PD-L1 antibody elicited the most pronounced immune-sensitization effect by promoting the infiltration and activation of CD8^+^ T cells. These results support the notion that CDKL1 functions as a suppressor of immune evasion in lung cancer.

## Conclusion

**Fig. 7:**
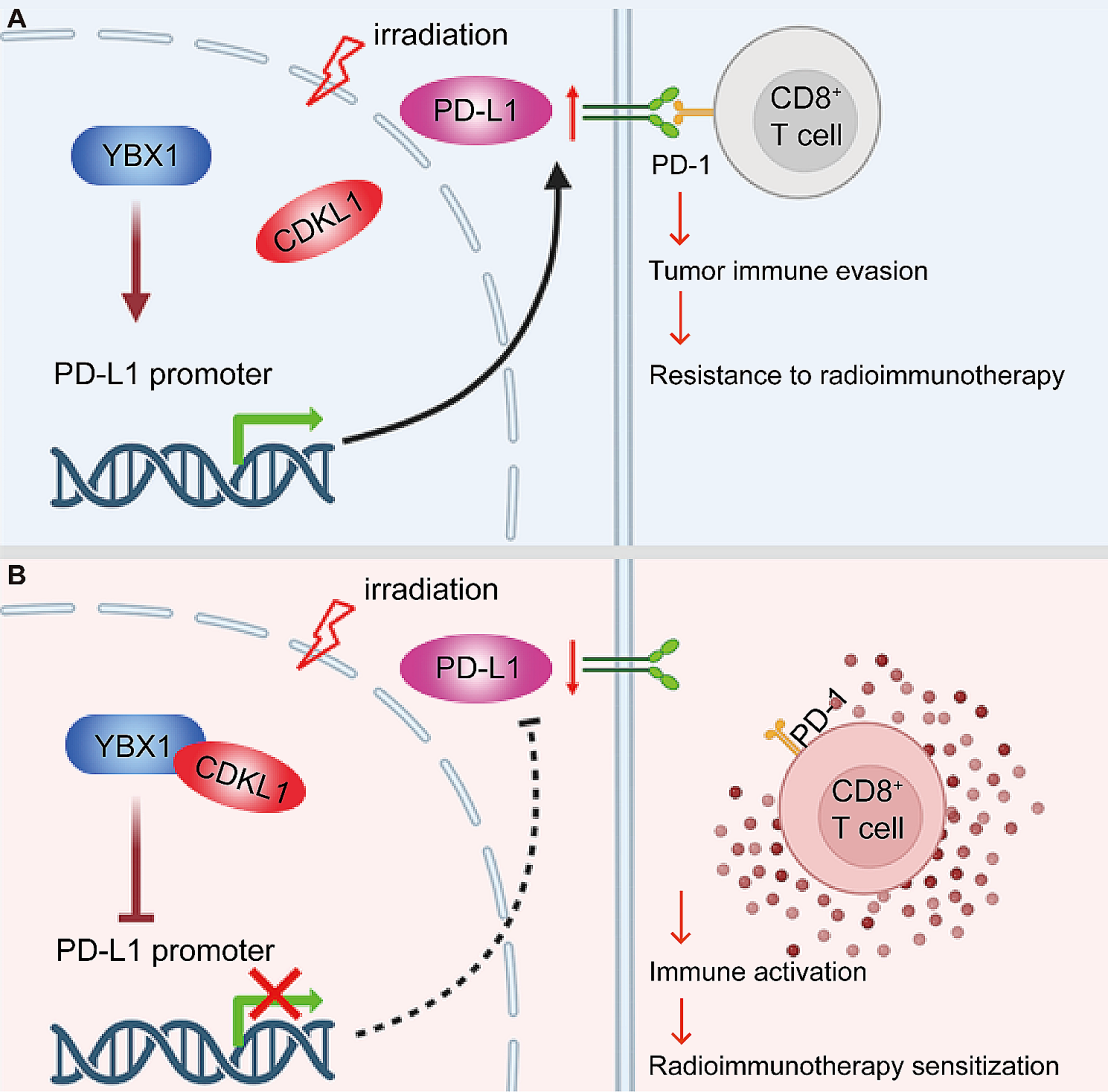
Model showing the mechanism through which CDKL1 is involved in immune evasion in lung cancer. (**A**) The absence of CDKL1 facilitates YBX1 binding to the PD-L1 promoter, thereby upregulating the expression of PD-L1. This, in turn, leads to the inactivation of CD8^+^ T cells and subsequent immune evasion, ultimately conferring resistance to radioimmunotherapy. (**B**) CDKL1 binds to YBX1 and suppresses the accumulation of YBX1 on the PD-L1 promoter, consequently downregulating the expression of PD-L1. This reduction in PD-L1 expression promotes the activation of CD8^+^ T cells, ultimately sensitizing lung cancer to radioimmunotherapy

In summary, our study provides a comprehensive understanding of the roles of CDKL1 in enhancing the radiosensitivity and inhibiting the immune evasion of lung cancer cells. Furthermore, we demonstrated that CDKL1 interacts with YBX1, which results in the inhibition of PD-L1 expression through the reduction of YBX1 binding to the PD-L1 promoter, thereby activating the CD8^+^ T-cell response and ultimately leading to radioimmunotherapy sensitization (Fig. 7). These findings provide novel molecular target and preclinical evidence for the development of radioimmunosensitization strategies to treat lung cancer.

### Electronic supplementary material

Below is the link to the electronic supplementary material.

Supplementary Material 1

## Data Availability

The analyzed datasets generated during the current study are available from the corresponding author on reasonable request.

## Abbreviations

CDKL1: cyclin-dependent kinase-like 1
PD-L1: programmed death-ligand 1
YBX1: Y box binding protein 1
NSCLC: non-small cell lung cancer
RT: radiotherapy
ChIP: chromatin immunoprecipitation
co-IP: Coimmunoprecipitation
