# Supplementary material for: CDKL1 potentiates the antitumor efficacy of radioimmunotherapy by binding to transcription factor YBX1 and blocking PD-L1 expression in lung cancer
Source: J Exp Clin Cancer Res. 2024 Mar 22;43:89. doi: 10.1186/s13046-024-03007-w (PMC10958935; doi:10.1186/s13046-024-03007-w)
Supplement: Supplementary file 1 — Supplementary Material 1 [file 13046_2024_3007_MOESM1_ESM.docx]

| **Genes** | **Sequences (5'--3')** |
| --- | --- |
| *PD-L1* (human) | F: TCACTTGGTAATTCTGGGAGC |
|  | R: CTTTGAGTTTGTATCTTGGATGCC |
| *PD-L1* (mouse) | F: ACTTGCTCATCTTCCTTTT |
|  | R: TTTACTATCACGGCTCCA |
| *CDKL1* (human) | F: GACAGATACCAAAGAGGGG |
|  | R: ACGGAATGTTTCGTGATG |
| *CDKL1* (mouse) | F: AAGGGCGATTTTCTTTAT |
|  | R: CCTATGGGGTAGTGTTCA |
| *GAPDH* (human) | F: AGAAGGCTGGGGCTCATTTG |
|  | R: AGGGGCCATCCACAGTCTTC |
| *GAPDH* (mouse) | F: TCAACGGCACAGTCAAGG |
|  | R: TTAGTGGGGTCTCGCTCC |

F, forward primer; R, reverse primer.

**Additional file 1: Table S1.** Sequences of primers used for qRT-PCR

| Genes | Primer | Sequences (5'--3') |
| --- | --- | --- |
| *PD-L1* | Primer 1 | F: TCTGACTTCTGACTTCGTT |
|  |  | R: TCTCCAAAGTCAGCCAAT |
|  | Primer 2 | F: TGTAAACCGAGGGCATTG |
|  |  | R: TAAAGCCATTTCCAGACT |
|  | Primer 3 | F: TGCTTTGATTGTCTTCTT |
|  |  | R: ACCGGTACACTCCAGGCT |
|  | Primer 4 | F: TCCCTTACTGAGGAGAAA |
|  |  | R: GAAACACCAAAGAAACAT |

F, forward primer; R, reverse primer.

**Additional file 2: Table S2.** Sequences of primers used for ChIP-PCR.
